# Monoacylglycerols Activate TRPV1 – A Link between Phospholipase C and TRPV1

**DOI:** 10.1371/journal.pone.0081618

**Published:** 2013-12-02

**Authors:** Peter M. Zygmunt, Anna Ermund, Pouya Movahed, David A. Andersson, Charlotte Simonsen, Bo A. G. Jönsson, Anders Blomgren, Bryndis Birnir, Stuart Bevan, Alain Eschalier, Christophe Mallet, Ana Gomis, Edward D. Högestätt

**Affiliations:** 1 Department of Laboratory Medicine, Lund University, Lund, Sweden; 2 Wolfson Centre for Age-Related Diseases, King's College London, London, United Kingdom; 3 Department of Neuroscience, Uppsala University, Uppsala, Sweden; 4 Clermont Université, Université d'Auvergne, Pharmacologie Fondamentale et Clinique de la Douleur, Laboratoire de Pharmacologie, Facultés de Médecine/Pharmacie, Clermont-Ferrand, France; 5 Inserm, U1107 Neuro-Dol, Clermont-Ferrand, France; 6 CHU Clermont-Ferrand, Service de Pharmacologie, Hôpital G. Montpied, Clermont-Ferrand, France; 7 Instituto de Neurociencias, Universidad Miguel Hernández-Consejo Superior de Investigaciones Científicas, Sant Joan d'Alacant, Spain; 8 Lund University Pain Research Centre, Lund University, Lund, Sweden; St. Joseph's Hospital and Medical Center, United States of America

## Abstract

Phospholipase C-mediated hydrolysis of phosphatidylinositol 4,5-bisphosphate generates diacylglycerol, inositol 1,4,5-trisphosphate and protons, all of which can regulate TRPV1 activity via different mechanisms. Here we explored the possibility that the diacylglycerol metabolites 2-arachidonoylglycerol and 1-arachidonoylglycerol, and not metabolites of these monoacylglycerols, activate TRPV1 and contribute to this signaling cascade. 2-Arachidonoylglycerol and 1-arachidonoylglycerol activated native TRPV1 on vascular sensory nerve fibers and heterologously expressed TRPV1 in whole cells and inside-out membrane patches. The monoacylglycerol lipase inhibitors methylarachidonoyl-fluorophosphonate and JZL184 prevented the metabolism of deuterium-labeled 2-arachidonoylglycerol and deuterium-labeled 1-arachidonoylglycerol in arterial homogenates, and enhanced TRPV1-mediated vasodilator responses to both monoacylglycerols. In mesenteric arteries from TRPV1 knock-out mice, vasodilator responses to 2-arachidonoylglycerol were minor. Bradykinin and adenosine triphosphate, ligands of phospholipase C-coupled membrane receptors, increased the content of 2-arachidonoylglycerol in dorsal root ganglia. In HEK293 cells expressing the phospholipase C-coupled histamine H_1_ receptor, exposure to histamine stimulated the formation of 2-AG, and this effect was augmented in the presence of JZL184. These effects were prevented by the diacylglycerol lipase inhibitor tetrahydrolipstatin. Histamine induced large whole cell currents in HEK293 cells co-expressing TRPV1 and the histamine H_1_ receptor, and the TRPV1 antagonist capsazepine abolished these currents. JZL184 increased the histamine-induced currents and tetrahydrolipstatin prevented this effect. The calcineurin inhibitor ciclosporin and the endogenous “entourage” compound palmitoylethanolamide potentiated the vasodilator response to 2-arachidonoylglycerol, disclosing TRPV1 activation of this monoacylglycerol at nanomolar concentrations. Furthermore, intracerebroventricular injection of JZL184 produced TRPV1-dependent antinociception in the mouse formalin test. Our results show that intact 2-arachidonoylglycerol and 1-arachidonoylglycerol are endogenous TRPV1 activators, contributing to phospholipase C-dependent TRPV1 channel activation and TRPV1-mediated antinociceptive signaling in the brain.

## Introduction

Since the discovery and cloning of the first transient receptor potential (TRP) ion channel in the *Drosophila* phototransduction pathway [1], [2], [3], 28 different *trp* genes have been identified in the mammalian genome [4], [5], [6]. Although being involved in a wide range of cell functions, a common feature of many TRP channels is their regulation by phospholipase C (PLC)-coupled surface receptors [7]. As a consequence of receptor activation PLC cleaves phosphatidylinositol 4,5-bisphosphate (PIP_2_) into diacylglycerol (DAG) and soluble inositol 1,4,5-trisphosphate (IP_3_), which all have complex actions in cell signaling [8], [9]. The hydrolysis of the ester bond in PIP_2_ also generates protons [10], and DAG can be further metabolized by DAG lipase (DAGL) and monoacylglycerol lipase (MAGL) to yield 2-monoacylglycerols and free fatty acids [11].

Some TRP channels are directly activated by DAG or polyunsaturated fatty acids [12], [13], [14]. Removal of PIP_2_, protonation and protein kinase C (PKC)-mediated phosphorylation are other potential mechanisms linking PLC-coupled receptors to TRP channel gating [4], [5], [7], [10], [15], [16], [17], [18]. However, for many TRP channels, the mechanism of PLC-mediated ion channel activation remains elusive [5], [6], [7]. A critical role of DAGL in the activation of TRP in *Drosophila* phototransduction has been suggested, and it was speculated that 2-monoacylglycerols or saturated fatty acids could serve this messenger role [19], [20].

The capsaicin receptor TRPV1 is highly expressed in primary sensory neurons and mediates the proalgesic effect of inflammatory mediators that act on PLC-coupled surface receptors [16], [21]. TRPV1 is directly activated by unsaturated N-acylamines and certain lipoxygenase products, which are structurally related to 2-arachidonoylglycerol (2-AG) [22], [23], [24], [25], [26]. Although 2-AG is generally referred to as an endogenous cannabinoid receptor ligand [27], [28], there are indications in the literature that some biological effects of monoacylglycerols, including 2-AG, are mediated by TRPV1 [29], [30], [31]. However, a rapid metabolism of 2-AG and the formation of various TRPV1 active arachidonic acid (AA) metabolites [24], [32], [33], [34], [35], [36] could explain the activation of TRPV1 by 2-AG in these studies [29], [30], [31]. Thus, it remains to be shown that 2-AG as an intact molecule activates TRPV1 and contributes to the regulation of TRPV1.

In our study identifying anandamide (AEA) as an endovanilloid, we found that 2-AG produced minor TRPV1 currents [22]. Whether 2-AG as an intact molecule mediated this effect was not further examined. In the present study, we have addressed the possibility that the major DAG metabolites 2-AG and 1-AG, of which 2-AG originally was identified as an endogenous cannabinoid receptor ligand [27], as intact molecules are physiologically relevant activators of the capsaicin receptor TRPV1 and also take part in the PLC-TRPV1 signaling cascade.

Ion channel activity was assessed on heterologously expressed rat and human TRPV1, using the patch-clamp technique, and native TRPV1 present on sensory nerve endings in rodent mesenteric arterial segments, which are richly innervated by TRPV1 and calcitonin gene-related peptide (CGRP)-containing vasodilator nerve fibers [37], [38]. This latter bioassay of sensory nerve activity, in which the physiological readout is vasorelaxation, allows the study of TRPV1 in a native environment with no influence of cannabinoid CB1 and CB2 receptors [27], [39]. Importantly, this native multicellular bioassay is very sensitive as it involves a powerful physiological amplifier system (CGRP/cAMP), converting subtle TRPV1 activation into profound physiological responses that cannot be detected in single cell assays. Because it is well known that 2-AG and AA can be further metabolized to activators of TRPV1 or other TRP channels [24], [32], [33], [34], [35], [36], we also studied the enzymatic degradation of 2-AG in mesenteric arteries. Furthermore, we examined the biosynthesis of monoacylglycerols in dorsal root ganglia and human embryonic kidney 293 (HEK293) cells following exposure to pro-inflammatory mediators, acting on PLC-coupled surface receptors, and the involvement of monoacylglycerols as mediators of histamine-induced TRPV1 currents in HEK293 cells transiently expressing TRPV1 and the histamine H_1_ receptor. Finally, the possibility that inhibition of monoacylglycerol metabolism in brain produces TRPV1-mediated antinociception was tested.

## Results

### Monoacylglycerols activate sensory neurons

Rodent mesenteric arteries have been useful to study neuronal TRP channels in a native environment [22], [23], [38], [39], [40]. Initial experiments were therefore performed on this tissue, in which the physiological readout of TRPV1 activation is a robust CGRP-mediated vasorelaxation, to characterize the effects of monoacylglycerols and their possible metabolites on sensory nerve endings.

**Figure 1:**
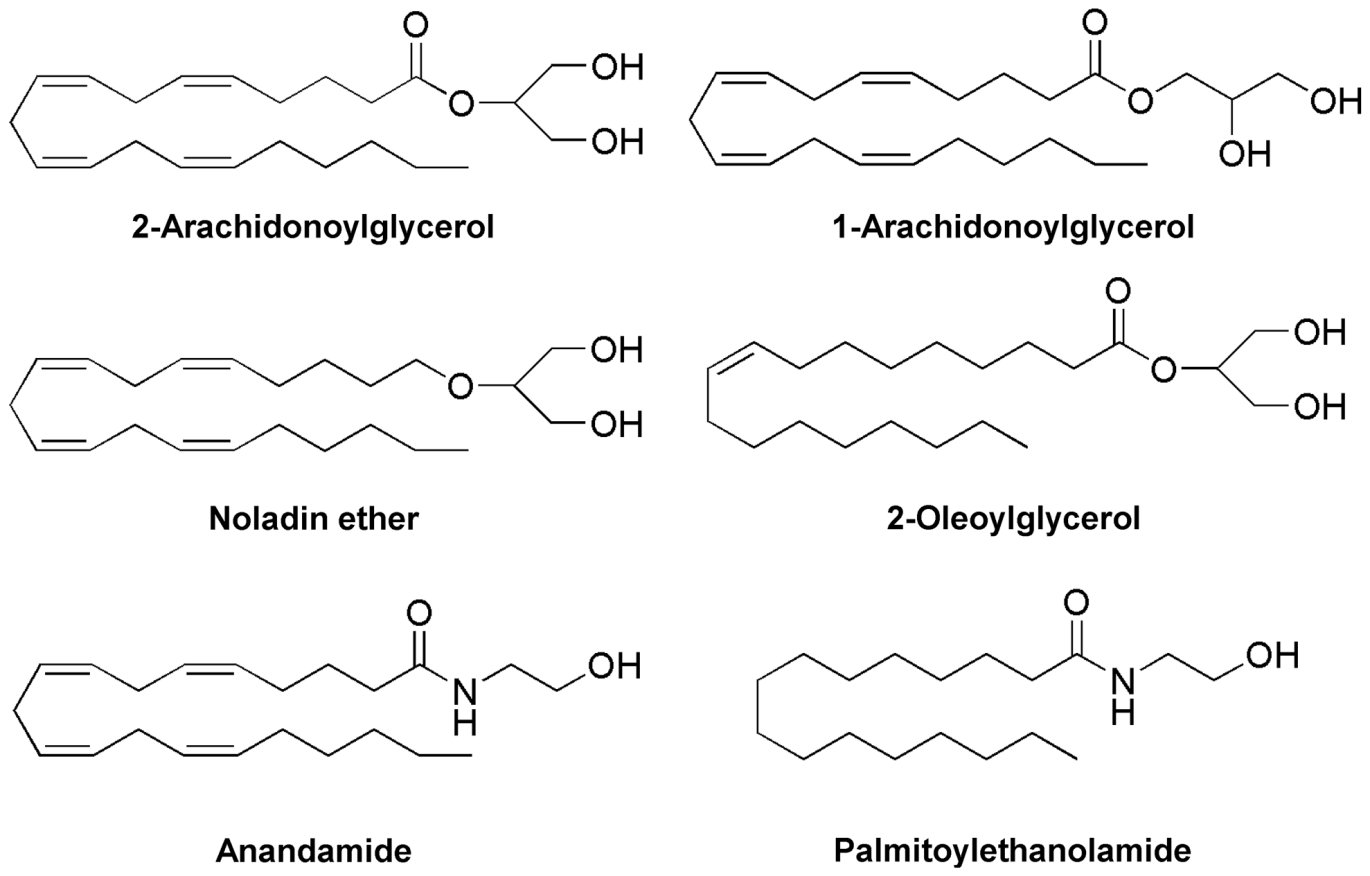
Chemical structures of endogenous monoacylglycerols and N-acylethanolamines. 2-Arachidonoylglycerol is a potent cannabinoid CB1 and CB2 receptor agonist that can undergo isomerization to 1-arachidonoylglycerol, which is not considered to have a role as an endocannabinoid. Noladin ether is a putative endocannabinoid that is much more stable to hydrolysis and enzymatic degradation than monoacylglycerols. Anandamide is an endocannabinoid and endovanilloid, whereas palmitoylethanolamide, which has no direct activity at cannabinoid CB1 and CB2 receptors, potentiates the effect of endocannabinoids and TRPV1-mediated responses.

**Figure 2:**
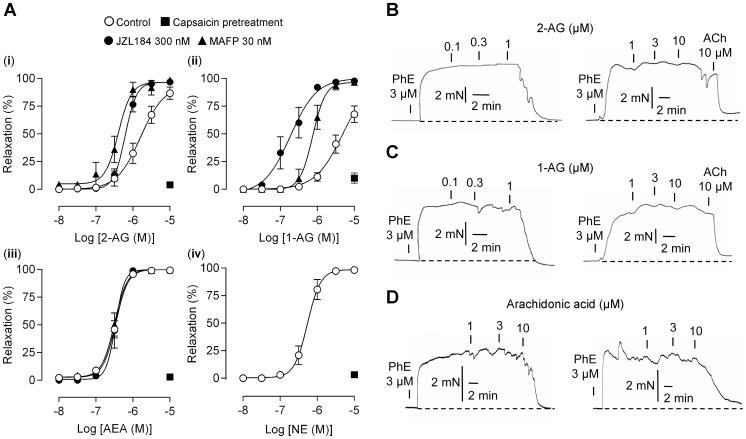
The monoacylglycerol lipase inhibitors MAFP and JZL184 potentiate 2-arachidonoylglycerol and 1-arachidonoylglycerol sensory nerve-mediated vasodilation. (A) Concentration-response curves for (i) 2-arachidonoylglycerol (2-AG), (ii) 1-arachidonoylglycerol (1-AG), and (iii) anandamide (AEA) in the presence of MAFP (n = 6–11), JZL184 (n = 6–8) or vehicle (n = 13–18). The area under the curve was larger in the presence than in the absence of MAFP (*P*<0.05, 2-AG; *P*<0.001, 1-AG) or JZL184 (*P*<0.05, 2-AG; *P*<0.001, 1-AG). (iv) Concentration-response curves for noladin ether (NE), the stable analog of 2-AG (n = 6–8). The vasodilation evoked by 2-AG, 1-AG, AEA and NE but not arachidonic acid were abolished by capsaicin pretreament (A–D). Traces show vasodilator responses to (B) 2-AG, (C) 1-AG and (D) arachidonic acid after pretreatment with vehicle (left) or capsaicin (right) to cause desensitization and/or neurotransmitter depletion of sensory nerve endings in rat mesenteric arteries. Subsequent application of acetylcholine (ACh) was used to confirm that arteries were able to respond with vasorelaxation (mediated by endothelium-derived hyperpolarising factor; [83]). The capsaicin pretreatment consisted of a 30 min exposure to 10 µM capsaicin followed by washout of capsaicin. The arterial segments were submaximally contracted with phenylephrine (PhE) before addition of the test drugs. The dashed line shows the basal tension level before addition of PhE. MAFP (30 nM) was present in experiments with 2-AG (B) and 1-AG (C). Control experiments were performed in the presence of vehicle (0.1% ethanol). Data are expressed as mean ± s.e.m.

The monoacylglycerols 2-AG (Figure 1) and 1-AG (Figure 1), and the N-acylethanolamine AEA (Figure 1) induced concentration-dependent vasorelaxation in rat mesenteric arteries (Figure 2). The related monoacylglycerol 2-oleoylglycerol (2-OG; Figure 1) at 10 µM induced only minor vasorelaxation (9±2%, n = 6). MAGL is a key enzyme in the elimination of monoacylglycerols [41], and we therefore tested the effect of the MAGL inhibitors methylarachidonoyl-fluorophosphate (MAFP) and 4-nitrophenyl 4-(dibenzo[d][1], [3]dioxol-5-yl(hydroxy)methyl)piperidine-1-carboxylate (JZL184) at concentrations of 30 and 300 nM, respectively [42], [43]. Incubation with these inhibitors enhanced the relaxation induced by 2-AG and 1-AG, but not AEA (Figure 2). Higher concentrations of the enzyme inhibitors could not be tested because they produced a capsaicin-insensitive vasorelaxation *per se*. Pretreatment with capsaicin (10 µM) almost abolished the vasodilator responses to 10 µM of 2-AG, 1-AG or AEA (n = 4–6, Figure 2). Noladin ether (Figure 1), a hydrolytically stable analog of 2-AG [44], [45], also elicited a concentration-dependent vasorelaxation that was prevented by capsaicin-pretreatment (n = 6–8, Figure 2).

As 2-AG and 1-AG are metabolized to AA and glycerol, we tested if these metabolites could mimic the action of the monoacylglycerols. While glycerol (10 µM) was inactive (0.1±0.1%, n = 5), AA at concentrations above 1 µM was able to produce a vasorelaxation, but this response was unaffected by capsaicin pretreatment in contrast to the vasodilator responses to 2-AG and 1-AG (Figure 2); the vasodilator response to the highest AA concentration tested (10 µM) was 58±17% and 61±18% in arteries pretreated with capsaicin and vehicle, respectively (n = 5).

The membrane permeable DAG analog 1-oleoyl-2-acetyl-sn-glycerol (OAG) at a concentration of 10 µM failed to induce vasorelaxation compared to its vehicle (OAG: 7±2%; vehicle 7±4%; n = 5).

### Metabolism of monoacylglycerols

**Figure 3:**
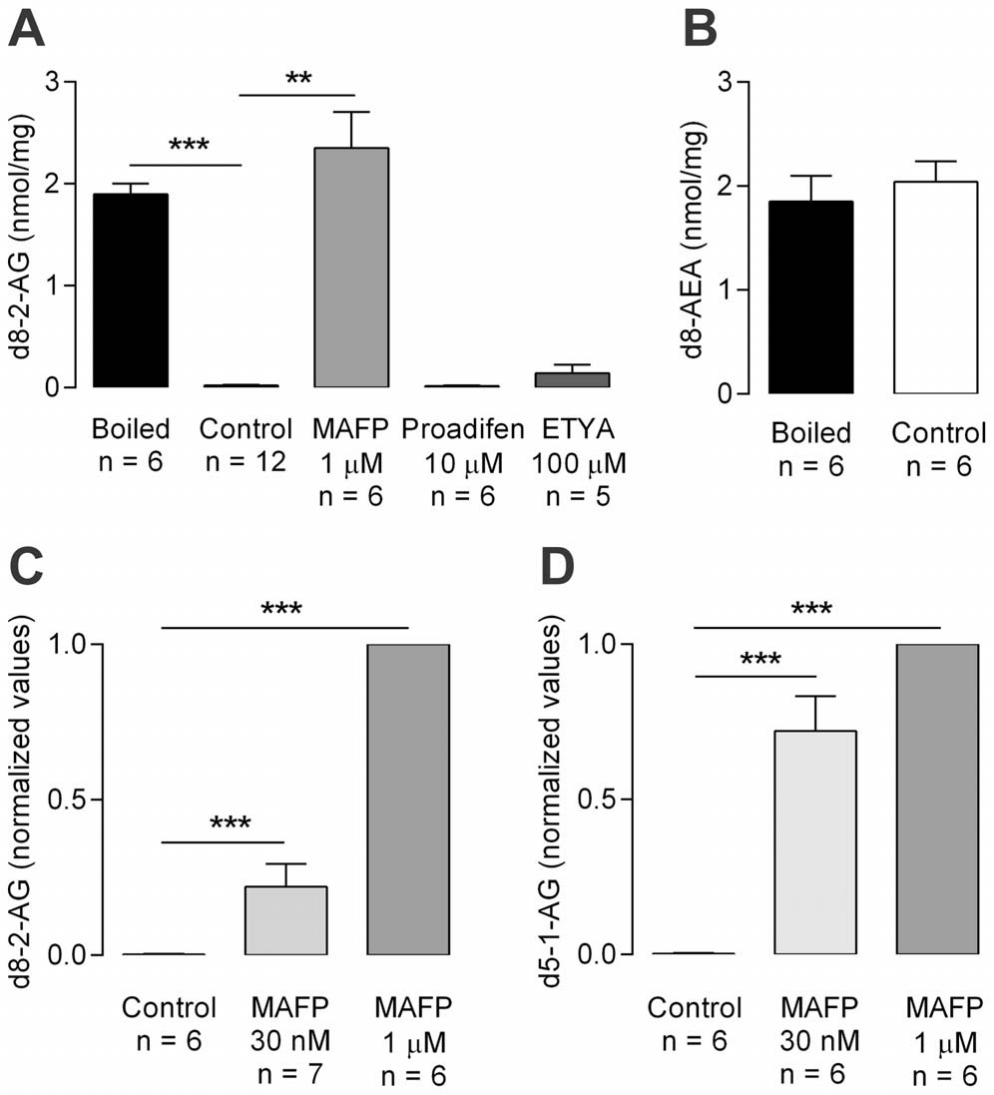
2-arachidonoylglycerol and 1-arachidonoylglycerol, but not anandamide, are metabolized in rat mesenteric artery homogenates. Deuterium(d)-labeled compound at a concentration of 1 µM were each added to the arterial homogenates and the amounts of d8-2-arachidonoyl glycerol (d8-2-AG), d8-anandamide (d8-AEA) and d5-1-arachidonoyl glycerol (d5-1-AG) remaining after 20 min were measured by mass-spectrometry. Enzymatic degradation was confirmed by comparing the amounts of d8-2-AG (A) and d8-AEA (B) in intact (Control) and boiled homogenates. The MAGL inhibitor methylarachidonoyl-fluorophosphonate (MAFP) inhibited the degradation of d8-2-AG (A–C) and d5-1-AG (D). The cytochrome P450 inhibitor proadifen and eicosatetraynoic acid (ETYA), a general inhibitor of arachidonic acid metabolism, did not affect the degradation of d8-2-AG (A). Control experiments were performed in the presence of vehicle (0.1% ethanol) for MAFP, proadifen and ETYA. Data are expressed as mean ± s.e.m. The number of independent experiments (n) is given in the figure. Kruskal-Wallis one-way ANOVA followed by Dunn's *post hoc* test was used for comparing groups of data. **P<0.01, ***P<0.001.

The above experiments indicated a pronounced metabolism of 2-AG and 1-AG in mesenteric arteries. We therefore characterized possible enzymatic pathways involved in the metabolism of the monoacylglycerols in this tissue. We found that d8-2-AG and d5-1-AG were extensively metabolized in homogenates of rat mesenteric arteries. After 20 min incubation (37°C) with d8-2-AG and d5-1-AG (each 1 µM), these monoacylglycerols almost disappeared from the homogenate (Figure 3A), whereas the level of d8-AEA was similar in untreated and boiled homogenates (Figure 3B). The monoacylglycerol lipase inhibitor MAFP prevented the metabolism of d8-2-AG and d5-1-AG (Figure 3C and D). Since MAFP is also an inhibitor of fatty acid amide hydrolase (FAAH), which is capable of metabolizing 2-AG [41], we performed additional experiments in tissue homogenates from FAAH knock-out mice. An MAFP-sensitive metabolism of d8-2-AG was also observed in homogenates of mesenteric arteries from such animals; the content of d8-2-AG was 4742±143 and 1440±794 pmol/mg protein in the presence and absence of 1 µM MAFP, respectively (P<0.05; n = 4). The general inhibitor of AA metabolism eicosatetraynoic acid (10 µM), targeting both cyclooxygenases and lipoxygenases, and the selective cytochrome P450 inhibitor proadifen (100 µM) did not affect the metabolism of d8-2-AG in homogenates of rat mesenteric arteries (Figure 3A).

### Biosynthesis of monoacylglycerols in dorsal root ganglia

**Figure 4:**
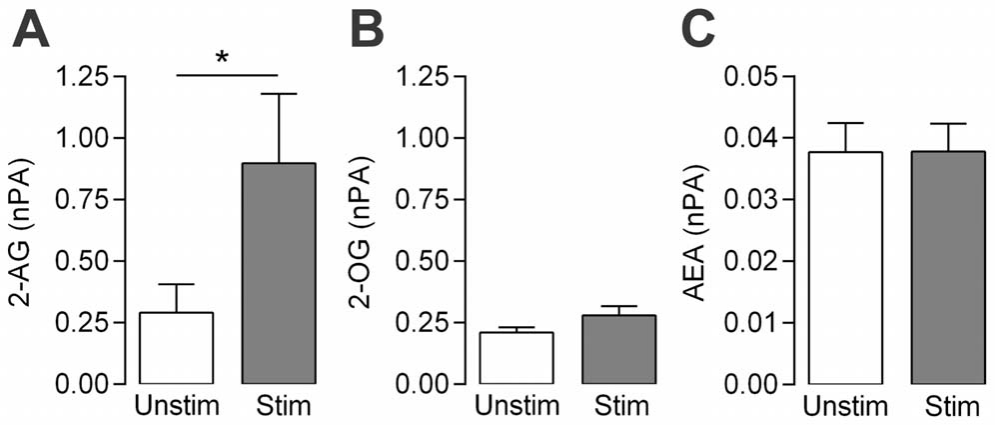
Bradykinin and ATP selectively increase the content of 2-arachidonoylglycerol in rat dorsal root ganglia. Isolated dorsal root ganglia from newborn rats were incubated with a mixture of 10 µM bradykinin and 1 mM ATP (Stim), or vehicle (Unstim) for 2 min and the contents of 2-arachidonoylglycerol (2-AG, A), 2-oleoylglycerol (2-OG, B) and anandamide (AEA, C) determined by mass-spectrometry. Y-axis indicates normalized peak area (nPA). Data are presented as mean ± s.e.m. (n = 6). Paired Student's *t*-test on log transformed values was used for comparing groups of data. *P<0.05

In order to provide direct evidence of the formation of monoacylglycerols in the nervous system, we used mass-spectrometry to quantify the levels of 2-AG and 2-OG in dorsal root ganglia from newborn rats. The basal level of 2-AG (440±180 pmol/mg protein) was approximately 30 times higher than that of AEA (15±4.2 pmol/mg protein). As it is well known that different G protein-coupled receptors, such as bradykinin B2 and purinergic P2Y receptors can sensitize sensory neurons, we used a mixture of bradykinin (10 µM) and ATP (1 mM) to stimulate the isolated dorsal root ganglia. Co-stimulation with these inflammatory mediators for 2 min caused an approximately 3-fold increase in the content of 2-AG, whereas the levels of 2-OG and AEA were unaffected (Figure 4).

### Monoacylglycerols activate TRPV1

**Figure 5:**
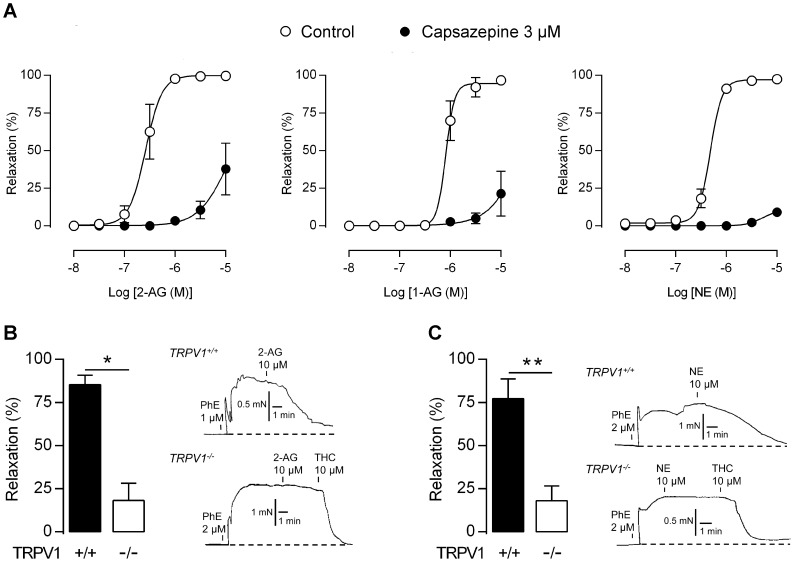
2-Arachidonoylglycerol, 1-arachidonoylglycerol and the stable 2-arachidonoylglycerol analog noladin ether activate native TRPV1. (A) Concentration-response curves for 2-arachidonoylglycerol (2-AG), 1-arachidonoylglycerol (1-AG) and noladin ether (NE) in the presence and absence of the TRPV1 antagonist capsazepine. Control experiments were performed in the presence of vehicle (0.1% ethanol). The MAGL inhibitor methylarachidonylfluorophosphonate (30 nM) was present in all experiments with 2-AG and 1-AG. The area under the curve was smaller in the presence than in the absence of capsazepine for all three lipids (*P*<0.01). Data are expressed as mean ± s.e.m. (n = 5–8).Vasodilator effect of 10 µM 2-AG (B) and NE (C) in isolated mesenteric arterial segments from TRPV1 knock-out (TRPV1^−/−^) and wild-type (TRPV1^+/+^) mice (n = 5). While 2-AG and NE produced only small responses in TRPV1^−/−^ mice, subsequent application of the TRPA1 activator Δ^9^-tetrahydrocannabinol (THC) elicited an almost complete vasorelaxation, amounting to 90±2.5% and 94±2.0%, respectively (traces). The dashed lines show the basal tension level before addition of phenylephrine (PhE). Data are expressed as mean ± s.e.m.. *P<0.05, **P<0.01.

In the presence of inhibitors of MAGL, the TRPV1 antagonist capsazepine (3 µM) almost abolished the vasorelaxation induced by 2-AG and 1-AG in rat mesenteric arteries (Figure 5A). Capsazepine similarly suppressed the vasodilator responses to noladin ether (Figure 5A). At a concentration of 10 µM, 2-AG and noladin ether produced robust vasorelaxation in mesenteric arteries from wild-type mice. These responses were substantially reduced in TRPV1 knock-out animals (Figure 5B and C).

**Figure 6:**
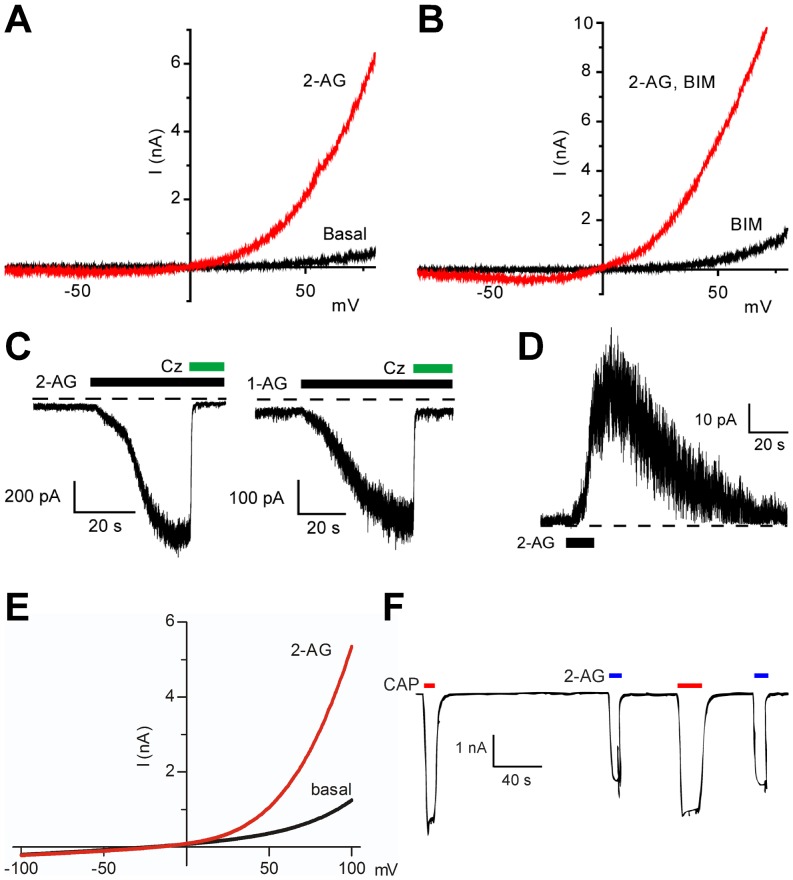
2-Arachidonoylglycerol and 1-arachidonoylglycerol activate heterologously expressed rat and human TRPV1. Current-voltage relationships after application of vehicle (Basal) or 2-arachidonoylglycerol (2-AG, 10 µM) to rat TRPV1-expressing CHO cells in the absence (A) and presence (B) of the protein kinase C inhibitor bisindoylmaleimide IV (BIM; 10 µM). Each current-voltage curve is representative of 3 cells. (C) Current traces showing responses to 10 µM 2-AG (left) and 10 µM 1-arachidonoyl glycerol (1-AG, right) in rat TRPV1-expressing CHO cells at a holding potential of −60 mV. Application of 10 µM of the TRPV1 antagonist capsazepine (Cz) immediately reversed the inward currents. (D) Excised inside-out patches from rat TRPV1-expressing CHO cells responded to 10 µM 2-AG with robust outward currents at a membrane potential of +60 mV. (E) Current-voltage relationships before and after application of 2-AG (10 µM) in HEK293 cells stably expressing human TRPV1. (F) Inward currents in HEK293 cells transiently expressing human TRPV1 at a holding potential of −50 mV. Traces show inward currents elicited by 2 µM capsaicin (CAP) and 10 µM 2-AG in a calcium free solution. Exposure to capsaicin and 2-AG a second time produced currents of similar magnitude. Data are presented as mean ± s.e.m. Kruskal-Wallis one-way ANOVA followed by Dunn's *post hoc* test was used for comparing groups of data. **P<0.01, ***P<0.001.

To obtain further evidence of TRPV1 as a molecular target for 2-AG and 1-AG, we performed electrophysiological experiments with heterologously expressed TRPV1. In CHO cells stably expressing rat TRPV1, 2-AG (10 µM) induced an outwardly rectifying current with a reversal potential of −0.5±1 mV (n = 4; Figure 6A). 2-Arachidonoylglycerol failed to evoke currents in untransfected CHO cells. The current responses to 2-AG were unaffected by the protein kinase C inhibitor bisindoylmaleimide IV (10 µM; Figure 6B). The TRPV1 blocker capsazepine (10 µM) completely inhibited the inward current induced by 2-AG and 1-AG (99±2% and 101±1% inhibition, respectively; n = 3; Figure 6C). Application of 2-AG (10 µM) to the cytoplasmic side of isolated inside-out membrane patches from TRPV1-expressing CHO cells evoked marked channel activity with a rapid onset at a membrane potential of +60 mV, consistent with a membrane-delimited site of action (Figure 6D).

In HEK293 cells stably expressing human TRPV1, 2-AG (10 µM, Figure 6E) induced whole cell currents, whereas the vehicle (0.1% ethanol) did not evoke currents (n = 3). At +100 mV, 2-AG increased the average current by 4.10±1.78 nA (n = 5), whereas non-induced cells did not respond to 2-AG (n = 9). In HEK293 cells transiently expressing the human TRPV1, 2-AG (10 µM) induced robust inward currents at a holding potential of −50 mV (Figure 6F). Approximately 50% of the cells that responded to capsaicin (2 µM) also responded to 10 µM 2-AG (20 out of 41 cells), which is most likely due to greater variations in TRPV1 expression levels when using transient transfection. The inward current evoked by 2-AG in these cells amounted to 44±8% of that elicited by capsaicin (n = 20).

### 2-AG contributes to phospholipase C-mediated regulation of TRPV1

To test if endogenous 2-AG and 1-AG activate TRPV1, we performed whole-cell patch clamp recordings in HEK293 cells co-expressing rat TRPV1 and canonical Gq/11-coupled histamine H1 receptors (H_1_R). Stimulation of the H_1_R-Gq/11-PLC pathway results in the synthesis of IP_3_ and DAG, the latter being converted by DAGL to 2-AG, which is then degraded to AA by MAGL [46], [47].

**Figure 7:**
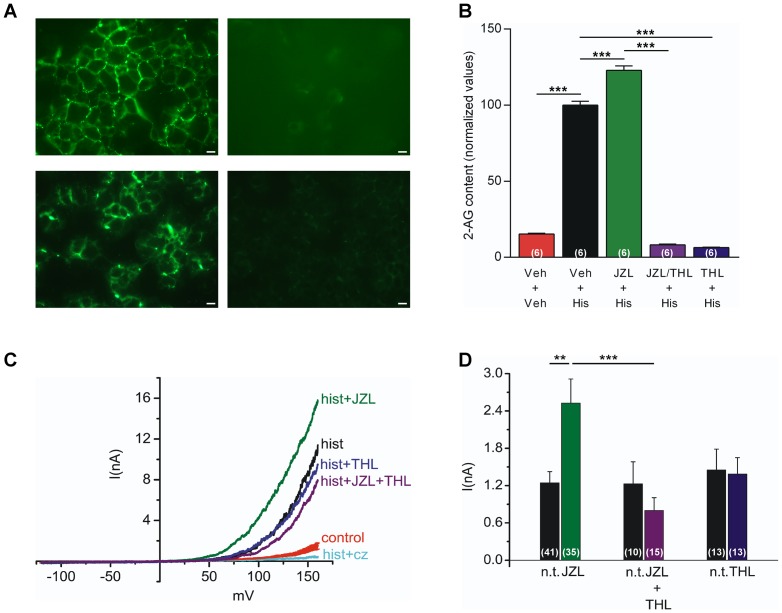
Contribution of 2-arachidonoylglycerol to TRPV1 activation evoked by stimulation of phospholipase C-coupled histamine H_1_ receptors in HEK293 cells. (A) Monoacylglycerol lipase (MAGL) expression in HEK293 cells, as shown by immunocytochemistry. Upper and lower left images show staining with human MAGL antibodies from Abcam and Pierce Biotechnology, respectively. Right hand images were obtained after incubation of the primary antibodies with respective blocking peptide. (B) Histamine (His, 100 µM) increased the content of 2-arachidonoylglycerol (2-AG) in HEK293 cells transiently expressing the phospholipase C-coupled histamine H_1_ receptor. Incubation (30 min) with the MAGL inhibitor JZL184 (JZL, 10 µM) further increased the 2-AG content, whereas the diacylglycerol lipase (DAGL) inhibitor tetrahydrolipstatin (THL, 10 µM) prevented the histamine-induced 2-AG increase. The 2-AG content was normalized to the average 2-AG content induced by histamine in vehicle-treated HEK293 cells in each experiment. The results are presented as mean ± s.e.m (n = 6). One-way ANOVA followed by Bonnferroni's *post hoc* test was used for comparing groups of data. (C) Current-voltage relationships in HEK293 cells transiently co-expressing TRPV1 and the rat histamine H_1_ receptor before (red trace) and after (black trace) exposure to histamine 100 µM in the absence and presence of JZL184 (10 µM), THL (10 µM), JZL184 plus THL (each 10 µM) or Cz (10 µM). The control trace shows the average current with error bars obtained from 119 cells. (D) The mean current amplitude elicited by histamine at a test potential of +100 mV in non-treated (n.t.) cells and in cells treated (at least 20 minutes) with enzyme inhibitors. Data are presented as mean ± s.e.m (n = 6–8). Kruskal-Wallis one-way ANOVA followed by Dunn's *post hoc* test was used for comparing groups of data. **P<0.01, ***P<0.001.

Quantitative real-time PCR analysis detected messenger RNA encoding DAGLα (C_T_ value  = 26±0.1), DAGLβ (C_T_ value  = 26±0.1) and MAGL (C_T_ value  = 31±0.1) in HEK293 cells. The C_T_ value for the house-keeping gene ubiquitin C was 18±0.1. Constitutive MAGL protein expression in HEK293 cells was also validated by immunocytochemistry (Figure 7A). The basal content of 2-AG in mock-transfected HEK293 cells was low and unaffected by a 3 min histamine (100 µM) exposure (n = 6, data not shown). However, histamine produced a more than 6-fold increase in the 2-AG content in HEK293 cells transiently expressing rat H_1_R (Figure 7B). The MAGL inhibitor JZL184 (10 µM) further increased and the DAGL inhibitor tetrahydrolipstatin (THL, 10 µM) [46], [47], [48] prevented the histamine-induced increase in the 2-AG content (Figure 7B).

As shown in Figure 7C, histamine (100 µM) induced large and reversible whole cell currents in HEK293 cells co-transfected with rat *trpv1* and *h1r*, but not in cells transfected with rat *h1r* only (Figure S1). Histamine-induced currents were instantly inhibited by capsazepine (10 µM; n = 4; Figure 7C).

The currents evoked by histamine were enhanced in HEK293 cells incubated with JZL184 (10 µM) compared to non-treated cells (Figure 7C and D). HEK293 cells were also incubated with THL (10 µM) [46], [47], [48] alone or in combination with JZL184 (10 µM). The mean amplitude of the recorded currents at +100 mV are summarised in Figure 7D. The averaged current in HEK293 cells incubated with JZL184 (2.5±0.39 nA, n = 35) was significantly enhanced compared with non-treated cells (1.25±0.18 nA, n = 41). The incubation with both JZL184 and THL significantly reduced the current amplitude to 0.8±0.2 nA (n = 15) compared with HEK293 cells incubated with JZL184 alone. There was no significant difference between the histamine-induced current in HEK293 cells treated with JZL184 plus THL (0.8±0.2 nA, n = 15) and non-treated cells (1.23±0.36, n = 10). The histamine-induced currents in HEK293 cells treated with THL (1.38±0.27 nA, n = 13) were similar to non-treated cells (1.45±0.33 nA, n = 13).

To rule out the possibility that the treatment with the enzyme inhibitors exerted their effects by interacting with TRPV1, we performed experiments as above, using capsaicin (0.1 µM) instead of histamine to activate TRPV1. There was no significant difference between capsaicin-evoked currents in HEK293 cells incubated with JZL184 (9.7±0.9 nA, n = 19) and non-treated cells (8.4±0.8 nA, n = 35), between HEK293 cells incubated with THL (6.2±1 nA, n = 10) and non-treated cells (5.9±1.4, n = 9), and between HEK293 cells incubated with JZL184 plus THL (5.8±0.9 nA, n = 11) and non-treated cells (5.9±1.7 nA, n = 7).

### Sensitization of TRPV1-mediated responses to 2-AG

**Figure 8:**
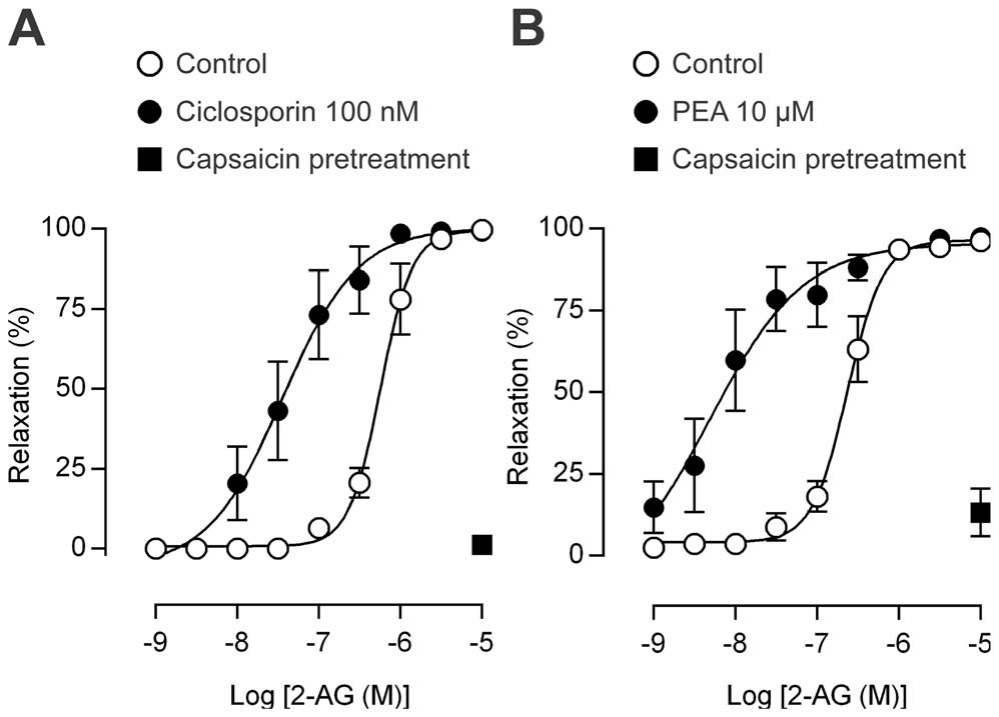
The calcineurin inhibitor ciclosporine and palmitoylethanolamide potentiate the sensory nerve-dependent vasodilator response to 2-arachidonoylglycerol. Effects of ciclosporine (A) and palmitoylethanolamide (PEA, B) on the vasorelaxation evoked by 2-arachidonoylglycerol (2-AG) in rat isolated mesenteric arterial segments. Pretreatment with 10 µM capsaicin almost abolished the 2-AG-induced relaxation in the presence of ciclosporine or PEA. The MAGL inhibitor methylarachidonylfluorophosphonate (30 nM) was present throughout. Data are presented as mean ± s.e.m (n = 6–8).

The calcineurin inhibitor ciclosporin and the endogenous “entourage” compound palmitoylethanolamide (PEA, Figure 1) both potentiate the sensitivity of TRPV1 to chemical stimulation [49], [50], [51]. We therefore tested whether the effect of 2-AG on TRPV1 is subject to a similar modulation. In rat mesenteric arteries, ciclosporin (100 nM) and PEA (10 µM) enhanced the 2-AG-induced vasorelaxation in the presence of MAFP (30 nM); the pEC_50_ for 2-AG was 7.5±0.3 and 8.1±0.3 in the presence and 6.3±0.1 and 6.6±0.1 in the absence of ciclosporin (P<0.001) and PEA (P<0.01), respectively (Figure 8). Capsaicin pretreatment almost abolished these 2-AG-induced responses (Figure 8), and, thus, additional non-sensory vasodilator mechanisms were not recruited by ciclosporin or PEA. Neither ciclosporin (n = 4; control: pEC_50_ 9.2±0.2, E_max_ 100±0.1; ciclosporin: pEC_50_ 9.3±0.1, E_max_ 100±0.1) nor PEA (n = 4; control: pEC_50_ 9.2±0.2, E_max_ 100±0.1; PEA: pEC_50_ 9.3±0.1, E_max_ 100±0.1) affected the relaxation induced by CGRP, ruling out a postjunctional site of action. In contrast to ciclosporin, the mechanism by which the “entourage” compound PEA potentiates TRPV1-mediated responses is unclear. We therefore tested the possibility that PEA inhibits the degradation of 2-AG. However, the content of d8-2-AG in homogenates of rat mesenteric arteries was not different in the presence (8.6±2.2 pmol/mg protein) and absence (10±2.4 pmol/mg protein) of 10 µM PEA (n = 4).

### JZL184-induced antinociception involves brain TRPV1

**Figure 9:**
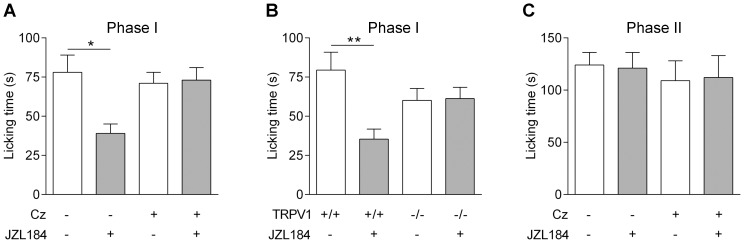
The monoacylglycerol lipase inhibitor JZL184 produces TRPV1-mediated antinociception. Intraplantar injection of formalin (2.5%) into the mouse paw produced a biphasic nociceptive response. (A) Intracerebroventricular (i.c.v.) injection of the monoacylglycerol lipase inhibitor JZL184 (10 nmol) 10 min before formalin injection produced antinociception in the first phase response and this effect was prevented by i.c.v co-administration of 10 nmol capsazepine (Cz). (B) The antinociceptive effect of JZL184 on the first phase of the formalin test was lost in TRPV1 knock-out mice. (C) The second nociceptive phase of the formalin response was unaffected by JZL184 (10 nmol) given i.c.v. 10 min after the intraplantar injection of formalin. Data are presented as mean ± s.e.m (n = 8–9). Kruskal-Wallis one-way ANOVA followed by Dunn's *post hoc* test was used for comparing groups of data.*P<0.05.

Based on findings that systemic administration of JZL184 increases endogenous levels of 2-AG in the brain [42], [43] and that TRPV1 activators when injected into brain produces antinociception [28], [52], [53], we tested the possibility that endogenous monoacylglycerols also produce TRPV1-mediated antinociception in the formalin test. Intracerebroventricular (i.c.v.) administration of JZL184 (10 nmol) 10 min before intraplantar formalin injection significantly suppressed the initial nocifensive behavior corresponding to the first phase of the formalin test (Figure 9A). The antinociceptive effect of JZL184 was prevented when JZL was co-injected with capsazepine (10 nmol) and absent in TRPV1 knock-out animals (Figure 9A and B). However, JZL184 did not affect the second phase of the formalin test neither when administered 10 min before (n = 9, data not shown) nor when administered 10 min after formalin injection (Figure 9C). Higher doses of JZL184 could not be tested due to limited solubility or vehicle effects.

## Discussion

**Figure 10:**
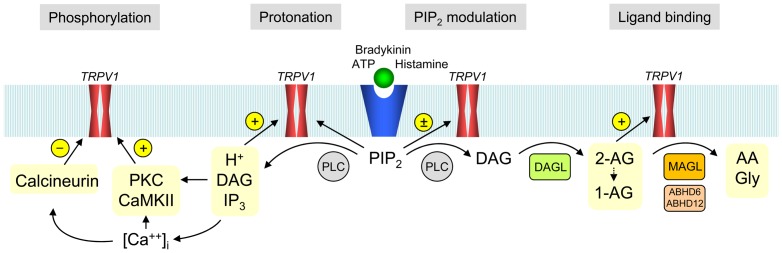
Diagram showing the contribution of 2-arachidonoyl glycerol to phospholipase C-dependent activation of TRPV1. Ligand-Gq-receptor interaction causes activation of phospholipase C (PLC), hydrolysis of phosphatidylinositol 4,5-bisphosphate (PIP_2_) and formation of diacylglycerol (DAG), which is further metabolized to 2-arachidonoylglycerol (2-AG) by diacylglycerol lipase (DAGL). 2-Arachidonoylglycerol undergoes a spontaneous acylmigration to yield 1-arachidonoylglycerol (1-AG). Both 2-AG and 1-AG directly activate TRPV1, and their effects are terminated by monoacylglycerol lipase (MAGL) and related enzymes, including the alpha/beta-hydrolases ABHD6 and ABDH12. Diacylglycerol indirectly regulates TRPV1 via protein kinase C (PKC)-dependent phosphorylation of the ion channel, whereas PIP_2_ has complex and opposing effects on TRPV1 channel gating. Hydrolysis of PIP_2_ also yields inositol 1,4,5-triphosphate (IP_3_), which via release of intracellular calcium causes activation of calcium-calmodulin-dependent protein kinase II (CaMK II). The phosphatase calcineurin dephosphorylates TRPV1. Protein phosphorylation, protonation, PIP_2_ modulation and binding of membrane-derived lipids provide an intricate system for fine-tuning of TRPV1 activity.

In the present study, we have used pharmacological and genetic strategies combined with biochemical and electrophysiological analyses to understand the cellular and molecular action of endogenous monoacylglycerols with regard to PLC-TRPV1 signaling in the nervous system. Our results demonstrate that the DAG metabolites 2-AG and 1-AG activate both native and heterologously expressed TRPV1 and suggest that these monoacylglycerols contribute to PLC-dependent TRPV1 channel activation (Figure 10), a possibility that may have previously been overlooked because of rapid metabolism of these lipids, recruitment of multiple activation pathways converging on TRPV1 and/or cannabinoid receptor-mediated functional antagonism in native tissues.

In contrast to AEA, 2-AG and 1-AG undergo extensive enzymatic degradation in rat mesenteric arteries, raising the possibility that TRPV1-active metabolites including those generated by cyclooxygenase [33], lipoxygenase [32], [34] or cytochrome P450 monoxygenase [35], mediated the effects of 2-AG and 1-AG in the present study. However, this is unlikely, because (i) 2-AG and 1-AG were more effective to elicit TRPV1-mediated vasorelaxations in the presence than in the absence of MAGL inhibitors, (ii) the two main metabolites of these monoacylglycerols, glycerol and AA, were either inactive (glycerol) or elicited a capsaicin-insensitive vasorelaxation (AA), (iii) vasorelaxation was recorded in the presence of the cyclooxygenase inhibitor indomethacin, (iv) inhibitors of cyclooxygenase, lipoxygenase or cytochrome P450 did not reduce the metabolism of 2-AG in tissue homogenates of rat mesenteric arteries and (v) 2-AG produced a rapid activation of TRPV1 in inside-out patches, which should exclude a contribution of any soluble and microsomal enzymes, such as cyclooxygenase, cytochrome P450 monoxygenase and most lipoxygenases. Taken together, our findings show that 2-AG and 1-AG activate TRPV1 as intact molecules.

Our metabolic and functional studies clearly indicate that an enzyme sensitive to MAFP and JZL184 is responsible for the metabolism of 2-AG and 1-AG. Neither of these inhibitors affected the TRPV1-mediated vasodilator response evoked by AEA and no metabolism of this N-acylethanolamine was detected in homogenates of rat mesenteric arteries. Furthermore, an MAFP-sensitive metabolism of 2-AG was observed in mesenteric arteries from FAAH knock-out mice. Finally, JZL184, at a concentration much lower than that needed to block rat FAAH, but close to the IC_50_ on rat MAGL [54], potentiated the vasodilator responses to 2-AG and 1-AG. Based on these findings, the enzyme responsible for metabolism of 2-AG and 1-AG in mesenteric arteries is most likely MAGL, the main 2-AG metabolizing enzyme in the brain [55]. Our findings together with previous reports [56], [57] suggest an important role for MAGL in terminating the action of these monoacylglycerols also in peripheral tissues.

The ester bond or the position of the acyl group does not seem to be crucial for TRPV1 activity, because noladin ether is also a potent activator of TRPV1 and 1-AG and 2-AG had similar potencies in our assay. Interestingly, 2-AG may undergo base-catalyzed acylmigration to yield 1-AG [58], [59]. As shown by Rouzer et al. (2002), acylmigration occurs with a half-life of 10 min or longer in buffer solutions at physiological pH. In the present study, the effects of 2-AG developed within seconds in the patch-clamp experiments and minutes in the vasodilator assay and, thus, it is unlikely that 1-AG formed in the aqueous biophase mediated these effects of 2-AG. However, when acylmigration occurs it could change the balance between the effects on TRPV1 and the cannabinoid CB1 receptor in systems that contain both these target proteins, because the cannabinoid CB1 receptor, in contrast to TRPV1, does not recognize 1-AG [60]. Thus, under conditions when 2-AG is the sole mediator in cannabinoid receptor signaling, acylmigration could influence the degree of CB1 receptor-mediated inhibition of TRPV1. This could be of importance both in the somatosensory nervous system and brain during, e.g., ischemia and inflammation, conditions under which base-catalyzed acylmigration would be expected to be reduced.

Dorsal root ganglion neurons are able to produce endogenous TRPV1 ligands, including products of 12-lipoxygenase [21], [24]. In the present study, we detected substantial amounts of 2-AG in rat dorsal root ganglia, and the level considerably exceeded those of AEA and other TRPV1 active endogenous N-acylethanolamines [23]. A mixture of the inflammatory mediators bradykinin and ATP, which were used to activate the PLC signaling pathway, increased the formation of 2-AG, but not 2-OG and AEA, in rat dorsal root ganglia. Such a difference is in line with the current view that 2-AG and AEA are regulated by distinct and independent biosynthetic pathways, and may indicate that the biosynthesis of individual species of monoacylglycerol could also be regulated differently [11], [61], [62], [63]. As neonatal rat dorsal root ganglia not only consist of neurons but also glial cells, we cannot pinpoint the cellular source of 2-AG. However, both these cell types have been reported to produce 2-AG and express TRPV1 [64], [65], [66], [67]. Regardless of the cellular source of 2-AG, the main metabolic pathway by which 2-AG is generated involves the hydrolysis of PIP_2_ into DAG and its further metabolism by DAGL to 2-AG [28], [46], [47].

Because DAG-mediated activation of PKC is one way by which PLC could modulate TRPV1 channel activity [15], [68], [69], we also considered that 2-AG might activate TRPV1 indirectly via PKC-mediated phosphorylation of TRPV1. However, in CHO cells expressing rat TRPV1, 2-AG elicited a characteristic TRPV1 current-voltage relationship, which was similar in the absence and presence of the PKC inhibitor bisindoylmaleimide IV. In addition, the robust currents evoked by 2-AG in inside-out patches indicate a membrane-delimited site of action independent of cytosolic enzymes including PKCε, the PKC isoform activated by bradykinin in nociceptors [68]. Furthermore, the cell permeable DAG analog OAG, which is an activator of PKC, could not mimic the action of 2-AG and 1-AG in rat mesenteric arteries, which is in line with recent findings that DAG cannot activate the purified TRPV1 reconstituted in lipid bilayers [70]. Taken together, these results indicate that 2-AG and 1-AG do not act indirectly via PKC-mediated phosphorylation of TRPV1, but rather suggest that these monoacylglycerols directly interact with this ion channel, as has been demonstrated for capsaicin and AEA [70].

We used HEK293 cells co-expressing the histamine H_1_ receptor and TRPV1 as a cellular test system to investigate the possibility that endogenous 2-AG can serve as a mediator of PLC-dependent activation of TRPV1. In support of this, the MAGL inhibitor JZL184 enhanced both the net 2-AG formation and the TRPV1-mediated current induced by histamine. However, we cannot exclude an effect of JZL184 on other monoacylglycerol degrading enzymes such as alpha/beta-hydrolases 6 and 12 [47], [54], [63]. Furthermore, the DAGL inhibitor THL abolished the histamine-induced 2-AG formation as well as the effect of JZL184 on the histamine-induced current. The reason why DAGL inhibition alone did not change basal histamine currents, while substantially reducing the 2-AG content, can only be speculated on, but one possibility could be that THL by increasing the levels of DAG maintained the histamine-induced TRPV1 activity via alternative pathways thereby compensating for the diminished 2-AG-mediated stimulation of TRPV1. Taken together, it is proposed that 2-AG acts in concert with PKC and other PLC-dependent signaling pathways to fine-tune TRPV1 channel activity (Figure 10).

The state of phosphorylation of TRPV1 is influenced by other proteins than PKC [71]. The ciclosporin-sensitive phosphatase calcineurin is one such protein, the inhibition of which prevents desensitization of capsaicin-induced currents in rat dorsal root ganglion neurons and TRPV1-expressing HEK293 cells as well as enhance calcium influx evoked by partial TRPV1 agonists in CHO cells stably expressing TRPV1 [49], [50], [71]. We were therefore curious to investigate the effect of the calcineurin inhibitor ciclosporin on 2-AG-evoked responses mediated by native TRPV1. Interestingly, we found that ciclosporin significantly potentiated the TRPV1-mediated vasorelaxation in mesenteric arteries disclosing an effect of this monoacylglycerol on TRPV1 in the nanomolar range. Likewise, the endogenous TRPV1 sensitizer PEA [51] displayed a similar synergistic effect in rat mesenteric arteries without interfering with the metabolism of 2-AG. These findings indicate that not only enzymatic degradation of 2-AG, but also posttranslational modifications of TRPV1 may influence the effect of 2-AG. Thus, local inflammation or other factors that affect these processes could switch 2-AG into an ultrapotent TRPV1 activator.

In the brain, 2-AG is established as a retrograde signaling molecule and TRPV1 is emerging as an important transduction protein involved in the regulation of synaptic signaling [28], [52], [72], [73]. The identification of JZL184 as a potent and selective MAGL inhibitor has provided a unique pharmacological tool to selectively elevate endogenous 2-AG levels *in vitro* and *in vivo* in order to understand the physiological role of this lipid messenger [47], [56], [63]. In the brain, the potent TRPV1 agonist AM404, a metabolite of paracetamol (acetaminophen), and the formation of the even more potent TRPV1 agonists olvanil and arvanil evoke TRPV1-mediated antinociception in the mouse formalin test [52], [72], [73], [74]. Using the same test in the present study, we found that intracerebroventricular injection of JZL184 in mice produced a substantial antinociceptive effect that was blocked by co-injection of capsazepine and absent in TRPV1 knock-out mice, suggesting that activation of TRPV1 by 2-AG is of physiological relevance. As in the case of AM404, the exact site of action within the CNS remains to be shown, but one possibility could be the midbrain region including rostral ventromedial medulla and periaqueductal gray, in which TRPV1 stimulation leads to activation of descending anti-nociceptive pathways [28], [53].

Interestingly, both phases of the formalin test are inhibited by intracerebroventricular administration of the TRPV1 activator AM404 [52], whereas in the present study JZL184 had no effect on the second phase of the formalin test even when injected 10 min after formalin injection to secure drug availability in case of rapid clearance or degradation of JZL184. However, a long lasting effect of JZL184 is expected, because this compound binds covalently to MAGL [42], [43]. Thus, these results could indicate that the first and second phases of the formalin test are under different supraspinal control by endogenous monacylglycerols. However, further investigations specifically exploring this possibility are warranted. In this context, it is interesting that JZL184 given systemically to mice produced antinociception also in the second phase of the formalin test [43], suggesting a local peripheral or spinal antinociceptive action of 2-AG. Whether inhibition of the first phase [43] was evoked at a supraspinal site, as described in the present study, or due to a local peripheral effect, as shown in the rat formalin test [56], is not known. Nevertheless, the present study has revealed that administration of the MAGL inhibitor JZL184 directly to the brain produces a TRPV1-dependent antinociceptive effect in the first phase of the mouse formalin test.

The majority of invertebrates do not express cannabinoid CB1 and CB2 receptors in contrast to TRP channels and metabolic pathways for monoacylglycerol biosynthesis and degradation [46], [75]. For example, a PLC-DAGL-TRP channel signaling axis is present in *Drosophila* phototransduction and 2-AG/TRPV1-like long-term depression of synaptic transmission exists in the leech, in which cannabinoid CB1 and CB2 receptors are absent [46], [75]. Although cannabinoid CB1 and CB2 receptors are considered as prime targets for 2-AG in mammalians, the ability of 2-AG to activate TRPV1 as well as bind to and increase the activity of GABA_A_ receptors [76] suggest multiple mechanisms by which this important signaling molecule can fine-tune neural activity, including long-term depression.

The results of the present study show that TRPV1 is activated by the endogenous monoacylglycerols 2-AG and 1-AG as intact molecules. Stimulation of PLC-coupled surface receptors triggered the biosynthesis of these monoacylglycerols in dorsal root ganglia and produces TRPV1 channel activation in HEK293 cells. Inhibition of MAGL in the brain evoked TRPV1-dependent antinociception in the formalin test. Together, this supports our idea that monoacylglycerols modulate synaptic activity by acting on TRPV1 in the nervous system. This may be of relevance for nociceptive neurotransmission and brain signaling mechanisms, including synaptic plasticity, in which 2-AG and TRPV1 have been proposed to play an important role [11], [28], [41], [52], [77], [78], [79]. Our finding may have general implications as PLC regulates many other TRP channels including those present in invertebrates.

## Materials and Methods

### Ethics Statement

The experiments were approved by the animal ethics committees in Auvergne, France (No. CE53-12 and CE09-08) and Malmö/Lund, Sweden (No. M82-10). The behavioural tests were conducted in accordance with the official edict presented by the French Ministry of Agriculture (Paris), the European Community Council Directive (86/609/EEC) and the International Association for the Study of Pain (IASP) guidelines for animal experiments [80].

### Recording of vasorelaxation

Wistar-Hannover rats (250 g) of female gender and C57 BL/6 mice (30 g) of either sex were purchased from Taconic (Ry, Denmark). TRPV1 gene knock-out mice and wild-type controls were obtained from Jackson laboratory (Bar Harbor, ME, USA). Animals were sacrificed by decapitation under CO_2_ anesthesia, and the mesenteric arterial bed flushed with physiological salt solution (composition in mM: NaCl 119, KCl 4.6, CaCl_2_ 1.5, MgCl_2_ 1.2, NaHCO_3_ 15, NaH_2_PO_4_ 1.2 and D-glucose 6). First and second order branches of the mesenteric artery were carefully dissected, and 2 mm long ring segments were suspended between two stainless steel wires in temperature-controlled tissue baths (37°C), containing aerated physiological salt solution (95% O_2_ and 5% CO_2_, pH 7.4), under a passive load of 2 mN (rat) or 1 mN (mouse). One of the wires was connected to an FT03 C force-displacement transducer (Grass Instruments, West Warwick, RI, USA) for isometric tension recording. After a 60 min equilibration period, vasorelaxation was studied in arterial segments submaximally contracted with phenylephrine. Increasing concentrations of test drugs (1 nM to 10 µM) were then added cumulatively to determine concentration-response relationships. All experiments were performed in the presence of indomethacin (10 µM) and N^ω^-nitro-L-arginine (300 µM) to reduce cyclooxygenase and nitric oxide synthase activity, respectively. Under these conditions, phenylephrine induces stable and long-lasting contractions, which were minimally affected by the vehicles used. Methylarachidonoyl-fluorophosphates, JZL184 and capsazepine were incubated with the preparation 40 min before addition of test drugs, whereas ciclosporin and PEA were added 5 min before 2-AG. Some arterial segments were pretreated with 10 µM capsaicin for 30 min to cause desensitization and/or neurotransmitter depletion of sensory nerve endings [81].

### Metabolism of deuterium-labeled monoacylglycerols and AEA

Mesenteric arteries were collected from rats and mice and homogenized in Tris buffer, composed of 10 mM Tris-HCl, 0.3 mM ascorbic acid and 1 mM EDTA 1.0 (pH 7.6). Homogenates were divided into aliquots of 250 µl and incubated with MAFP (30 nM and 1 µM), proadifen (100 µM) and eicosatetraynoic acid (10 µM) for 50 min or PEA (10 µM) for 5 min before addition of 1 µM of either d8-2-AG and d5-1-AG or d8-AEA. The reaction was stopped after 20 min with ice-cold acetone (1.25 ml), containing 0.3 mM ascorbic acid (antioxidant), and d8-AEA or d8-2-AG as internal standard. After centrifugation at 3000 rpm for 10 min at 5°C, the supernatant was collected in polypropylene tubes and evaporated in a vacuum centrifuge. The extraction residue was reconstituted in 100 µl methanol with 0.5% acetic acid and stored at −20°C until analyzed by liquid chromatography tandem mass-spectrometry (LC-MS/MS) for quantification of d8-2-AG, d5-1-AG and d8-AEA. The protein content of the pellet was determined with Coomassie (Pierce, Thermo Fisher Scientific, Rockford, IL) protein assay, using bovine serum albumin as a standard.

### Quantification of monoacylglycerols and AEA in dorsal root ganglia

Rat dorsal root ganglia from all spinal levels were dissected and homogenized in 250 µl ice-cold Tris buffer, containing MAFP (1 µM) to reduce enzymatic hydrolysis of monoacylglycerols and N-acylethanolamines. Ice-cold acetone (1.25 ml), supplied with 0.3 mM ascorbic acid and 0.1 µM d8-AEA as internal standard, was then added to the homogenates for extraction of lipids. The samples were prepared for mass-spectrometry analyses and protein quantification as described above.

Receptor-mediated formation of 2-AG, 2-OG and AEA was studied in dorsal root ganglia collected from neonatal rats. Ten pairs of dorsal root ganglia from each animal were divided between two test tubes, containing 200 µl HEPES buffer (composition in mM: HEPES 10, NaCl 140, KCl 5, CaCl_2_ 2, MgCl_2_ 2 and D-glucose 10; pH 7.4). After a 60 min equilibration period in a heating block (37°C), the two test tubes were exposed to either bradykinin (10 µM) plus ATP (1 mM) or vehicle for 2 min. The reaction was stopped by adding 1 ml ice-cold acetone, containing 0.3 mM ascorbic acid and 0.1 µM d8-AEA as internal standard. The tubes were centrifuged in an Eppendorf centrifuge at 13000 rpm for 30 min at 4°C. The supernatants were vacuum evaporated and the residues were reconstituted in 100 µl methanol with 0.5% acetic acid and stored at −20°C until analyzed by LC-MS/MS.

### Quantification of histamine-induced production of 2-AG in HEK293 cells

HEK293 cells grown to confluence in 75 cm^2^ flasks were transiently transfected with 19 µg pcDNA3 plasmid, encoding the rat histamine H_1_ receptor, or empty plasmid (mock-transfected), using lipofectamine 2000 (Invitrogen, Carlsbad, CA, USA) in a ratio of 1∶3 (µg cDNA/µl lipofectamine) in Opti-MEM (Invitrogen) for 4–6 hrs. Following incubation overnight in DMEM (Invitrogen), containing 10% FBS (Sigma) and 1% Glutamax (Invitrogen), cells were trypsinized and transferred to a 150 cm^2^ flask. Approximately 48 hours after the transfection was started, cells were washed twice with PBS (Invitrogen) and harvested with a cell scraper. The cell suspension was centrifuged at 1000 *g* and resuspended in 2.5 mL HEPES buffer.

Rat histamine H_1_ receptor gene-transfected HEK293 cells were incubated with vehicle (0.2% DMSO), JZL184 10 µM, THL 10 µM or JZL184 10 µM plus THL 10 µM for at least 30 min at 24°C. The cell suspensions were then divided into aliquots of 250 µl, and cells were stimulated with either vehicle (H_2_O) or histamine (100 µM) for 3 min at 24°C. The reaction was stopped with ice-cold acetone (1.25 ml), containing 0.3 mM ascorbic acid (antioxidant) and d5-AM404 as internal standard. After centrifugation at 3000 rpm for 10 min at 4°C, the supernatant was collected in polypropylene tubes and evaporated in a vacuum centrifuge. The extraction residue was reconstituted in 100 µl methanol with 0.5% acetic acid, centrifuged at 14000 rpm for 10 min at 4°C and the supernatant stored at −20°C until analyzed by mass-spectrometry. The experiments were replicated three times, each time in duplicates.

### Mass-spectrometry

A Perkin Elmer series 200 LC system with autosampler (Applied Biosystems, Norfolk, CT), coupled to an API 3000 LC-MS-MS (Applied Biosystems/MDS-SCIEX, Toronto, Canada), was used for the analysis. The column was a Genesis C8 (20×2.1 mm) with a particle size of 4 µm (Jones, Lakewood, CO). Aliquots of 5 µl were injected by the autosampler. The mobile phase was a water-methanol gradient, containing 0.5% acetic acid, and the initial mobile flow was 75% methanol. A linear gradient to 100% methanol was applied in 6 min. The mobile flow rate was 0.2 ml/min. The turbo ion spray interface was 370°C, the declustering potential was 40 volts and the collision energy was 35 volts. The analyses were performed in the positive ion multiple reaction monitoring mode and the mass fragments for detecting 2-AG/1-AG, d8-2-AG, d5-1-AG, 2-OG, AEA and d8-AEA were m/z 379.2/287.0, m/z 387.2/295.0, m/z 384.6/287.3, m/z 357.3/265.3, m/z 348.2/62.0 and m/z 356.4/63.0, respectively. The peak area ratios between analyte and internal standard were used for quantification (normalized peak areas). Since 2-AG is non-enzymatically converted to 1-AG, the content of 2-AG was estimated as the sum of 2-AG and 1-AG [58], [59]. The same approximation was used for quantification of d8-2-AG. The within day precisions were 10% and 8% for 2-AG, and 4% and 7% for AEA at 100 nM and 1000 nM, respectively.

### Immunocytochemistry

A suspension of HEK293 cells was smeared on a Superfrost Plus glass slide (Menzel-Gläser, Braunschweig, Germany), air-dried and fixed for 5 min with paraformaldehyde (2.0%) and picric acid (0.2%) in phosphate-buffered saline (pH 7.2). The slides were processed for demonstration of MAGL, using indirect immunofluorescence [82]. After pretreatment with BSA (1%) and Triton X-100 (0.2%) for two hours, the slides were incubated with affinity purified MAGL antibodies raised in rabbit (dilution 1∶100; Abcam, Cambridge, UK) or goat (dilution 1∶300; Pierce Biotechnology, Rockford, IL, USA) at room temperature overnight. The specimens were then incubated with the fluorescent secondary antibody (Alexa Fluor 488b goat anti-rabbit or donkey anti-goat from Molecular Probes, Eugene, OR, USA) at a dilution of 1∶800 for one hour. Control experiments were performed in the absence of the primary antibody, and by pre-incubation with surplus of blocking peptide (Abcam and Pierce Biotechnology). Digital photographs of the cells were obtained through a Nikon Eclipse TE2000-S microscope equipped with fluorescence optics with an attached digital camera and a computer equipped with the software NIS-elements BR 3.0 (Nikon). Photoshop 7.0 (Adobe) was used for processing of images.

### Real-time PCR

Quantitative real-time PCR (qPCR) was performed as previously described [82]. RNA was isolated from cultured HEK293 cells with Nucleospin RNA II Kit (Macherey-Nagel, Duren, Germany), and 1 µg of RNA was subjected to reverse transcription to cDNA. Real time fluorescence monitored PCR reactions were performed using the following TaqMan Gene Expression Assays (Applied Biosystems, Life Technologies, Carlsbad, CA): DAGLα (Hs00391374_m1), DAGLβ (Hs00373700_m1) and MAGL (Hs00200752_m1). Genes of interest were normalized to the reference gene Ubiquitin C (PrimerDesign, UK). Reactions were carried out in triplicate. C_T_ values below 37 were considered positive.

### Electrophysiology

#### Studies on rat TRPV1 expressed in CHO cells

Chinese hamster ovary (CHO) cells stably expressing rat TRPV1 were studied under voltage-clamp conditions, using an Axopatch 200B amplifier and pClamp 10.0 software (Axon Instruments, Sunnyvale, CA). Borosilicate glass pipettes (2–5 MΩ, 75–80% series resistance compensation) were filled with (in mM) KCl 140, CaCl_2_ 1, MgATP 2, EGTA 10 and HEPES 10; pH 7.4. The bath solution contained (in mM) NaCl 140, KCl 5, glucose 10, HEPES 10, EGTA 1 and MgCl_2_ 1; pH 7.4. Recordings from inside-out patches were performed using the bath solution both in the pipette and for superfusion. Drugs and solutions were applied by local superfusion, using a rapid solution changer (Bio-Logic, Claix, France). Stock solutions of monoacylglycerols were prepared in dimethyl sulfoxide.

#### Studies on human TRPV1 expressed in HEK293 cells

HEK293 cells were transfected with human TRPV1 cDNA, using lipofectamine (Invitrogen, Carlsbad, CA). After 24 hours, whole-cell currents were recorded at a holding potential of -50 mV using an Axopatch 200B amplifier and pClamp software (Axon Instruments, Sunnyvale, CA). The bath solution contained (in mM) NaCl 140, KCl 5, MgCl_2_ 2, D-glucose 10 and N-tris(hydroxymethyl) 10; pH 7.4. The pipette solution contained (in mM) CsCl 140, EGTA 5 and N-tris(hydroxymethyl) 10; pH 7.4. Drugs and solutions were applied by local superfusion.

Whole cell recordings in HEK293 cells stably expressing human TRPV1 were performed with an automated patch clamp platform, using a chip size of 2–3.5 MΩ (Port-a-Patch, Nanion Technologies GmbH, Munich, Germany) and an ECP10 amplifier (HEKA, Lambrecht, Germany). The bath solution contained (in mM): NaCl 140, KCl 5, MgCl_2_ 1, CaCl_2_ 2, D-glucose 10 and HEPES 10; pH 7.4 adjusted to with NaOH. The intracellular solution was composed of (in mM): CsCl 50, CsF 60, NaCl 10, EGTA 20 and HEPES 10; pH 7.2 adjusted with CsOH (∼285 mOsmol). Signals were sampled at 10–20 kHz and filtered at 2.9 kHz. Holding potential was −60 mV, and voltage ramps (−100 mV to 100 mV, 200–400 ms) were conducted every 3 s. 2-AG was applied directly to the extracellular chip solution.

#### Studies on HEK293 cells expressing the rat histamine H1 receptor and rat TRPV1

HEK293 cells were co-transfected with plasmid cDNA, encoding the rat histamine H_1_ receptor and rat TRPV1 fusioned to the green fluorescent protein (0.2 µg/ml and 1 µg/ml, respectively), using lipofectamine 2000 (Invitrogen) in a ratio of 1∶3 (µg cDNA/µl lipofectamine) for 4–6 hrs. Cells were trypsinized and replated on poly-lysine coated round coverslip 24 hrs after transfection, and the experiments were performed 48 hrs after transfection. Whole cell recordings were obtained, using 3–5 MΩ borosilicate glass capillary patch pipettes. Membrane currents were recorded with a Multiclamp 700B amplifier and ramp voltage clamp commands were applied, using pCLAMP software and a Digidata 1322A digitizer (Molecular Devices Corporation, Sunnyvale, CA, USA). Cells were held at a potential of −60 mV, and current-voltage (I–V) relations were obtained from voltage ramps from −120 mV to +160 mV with a duration of 350 ms applied every 3 s. Current was sampled at a frequency of 20 kHz. Series resistance compensation of >50% was used. Current amplitudes were measured at a holding potential of −100 and +100 mV. The standard bath solution contained the following (in mM): NaCl 140, KCl 3, CaCl_2_ 2.4, MgCl_2_ 1.3, HEPES 10 and glucose 10; pH 7.4 adjusted with NaOH. The pipette solution had the following composition (in mM): CsCl 135, MgCl_2_ 2, Na_2_ATP 5, EGTA 1 and HEPES 10, pH 7.4 adjusted with CsOH. Analysis was performed with pClamp9, WinASCD software (G. Droogmans, Katholieke Universiteit Leuven, Belgium) and Origin 7.5 (OriginLab Corporation, Northampton, MA, USA). JZL184 and THL were dissolved in dimethyl sulfoxide and stocks solutions were stored at −20°C. Further dilutions were made with the bath solution. Cell incubation with JZL184, THL or both was done for at least 20 min in a petri dish before cells were transferred to the recording chamber and superperfused with the solutions, also containing the corresponding drugs. Cells incubated with the vehicle dimethyl sulfoxide were interleaved with normal control experiments. Because we observed no vehicle effects, all control experiments were pooled.

### Nociceptive test

The antinociceptive effect of endogenous 2-AG was studied in the mouse formalin test. The test was performed in a quiet room and evaluated by a single investigator in a blinded manner. Treatments were randomized in blocks and the number of mice in each block corresponded to the number of treatments compared. Each mouse was exposed to only one treatment. Mice received an intraplantar injection of a 2.5% formalin solution (25 µl) into a hind paw. The time spent biting and licking of the injected paw was monitored during the two typical phases of the nociceptive response (phase I: 0–5 min; phase II: 15–40). JZL184 and/or capsazepine were injected intracerebroventricularly (left lateral ventricule) in mice under light isoflurane in volumes of 5 µl (5% DMSO and 2.5% Tween 80 in saline) 10 min before or after formalin injection.

### Calculations and statistics

Vasorelaxation was expressed as percentage reversal of the phenylephrine-induced contraction. The negative log molar concentration of drug that elicited half-maximal relaxation (pEC_50_) was calculated, using GraphPad Prism 6.0 software (GraphPad Software, Inc., La Jolla, San Diego, CA, USA). When concentration-response curves did not reach a plateau, the area under the curves (AUC) was determined (GraphPad Prism 6.0). When E_max_ was calculated to be higher than 100%, the E_max_ was locked to 100% in pEC_50_ calculations. The contents of lipids were related to the protein content in each sample and expressed as moles per mg protein. For comparison of 2-AG, 2-OG and AEA contents before and after treatment in isolated dorsal root ganglia and HEK293 cells, normalized peak areas were used. These values were obtained by dividing the peak area for the analytes with the peak area for the internal standard in the same sample. Data are presented as mean ± s.e.m., and n denotes the number of animals, cells or experiments. Mann-Whitney U test was used throughout for comparing groups of data unless stated otherwise (GraphPad Prism 6.0). Statistical significance was accepted when *P*<0.05.

### Drugs

Arachidonic acid (AA), 1-arachidonoylglycerol (1-AG), d5-1-arachidonoylglycerol (d5-1-AG), 2-arachidonoylglycerol (2-AG), d8-2-arachidonoylglycerol (d8-2-AG), methylarachidonoylfluorophosphonate (MAFP), 4-nitrophenyl 4-(dibenzo[d][1], [3]dioxol-5-yl(hydroxy)methyl)piperidine-1-carboxylate (JZL184) and palmitoylethanolamide (PEA) were purchased from Cayman Chemicals (Ann Arbor, MI, USA). Capsaicin and capsazepine were obtained from Tocris (Bristol, UK) and Sigma (St Louis, MO, USA). ATP, bisindoylmaleimide IV, bradykinin, Δ^9^-tetrahydrocannabinol, eicosatetraynoic acid, glycerol, histamine, 1-oleoyl-2-acetyl-sn-glycerol, 2-oleoylglycerol (2-OG), N^ω^-nitro-L-arginine, phenylephrine, proadifen, tetrahydrolipstatin (THL) and rat calcitonin gene-related peptide (CGRP) were purchased from Sigma. Indomethacin (Confortide®) was obtained from Dumex (Copenhagen, Denmark). The monoacylglycerols were delivered in acetonitrile, and the 2-AG and d8-2-AG stock solutions also contained approximately 10% of their respective regioisomers. Aliquots of the acetonitrile stock solution were evaporated under nitrogen and the content resuspended in ethanol or dimethyl sulfoxide, which was stored at −20°C before use. As shown by mass-spectrometry, the content of 2-AG and 1-AG remained stable under these conditions. Arachidonic acid, capsaicin, capsazepine, MAFP, Δ^9^-tetrahydrocannabinol, and monoacylglycerols and N-acylethanolamines were further diluted with ethanol or dimethyl sulfoxide immediately before the experiments. The final ethanol or dimethyl sulfoxide concentration never exceeded 1% and 0.2%, respectively.

## Supporting Information

Figure S1 **Histamine is unable to evoke whole cell currents in the absence of TRPV1.** Current-voltage relationships in HEK293 cells transiently expressing rat H_1_R (0.2 µg/ml) and GFP (1 µg/ml) proteins before (red trace) and after exposure to histamine (hist) 100 µM (black trace). Data were obtained by voltage ramps from −100 mV to +100 mV with a duration of 1.5 s applied every 5 s. Current was sampled at a frequency of 20 kHz. Series resistance compensation of >50% was used.
(TIF)
