# Supplementary figures and images for: Monoacylglycerols Activate TRPV1 – A Link between Phospholipase C and TRPV1
Source: PLoS One. 2013 Dec 2;8(12):e81618. doi: 10.1371/journal.pone.0081618 (PMC3847081; doi:10.1371/journal.pone.0081618)

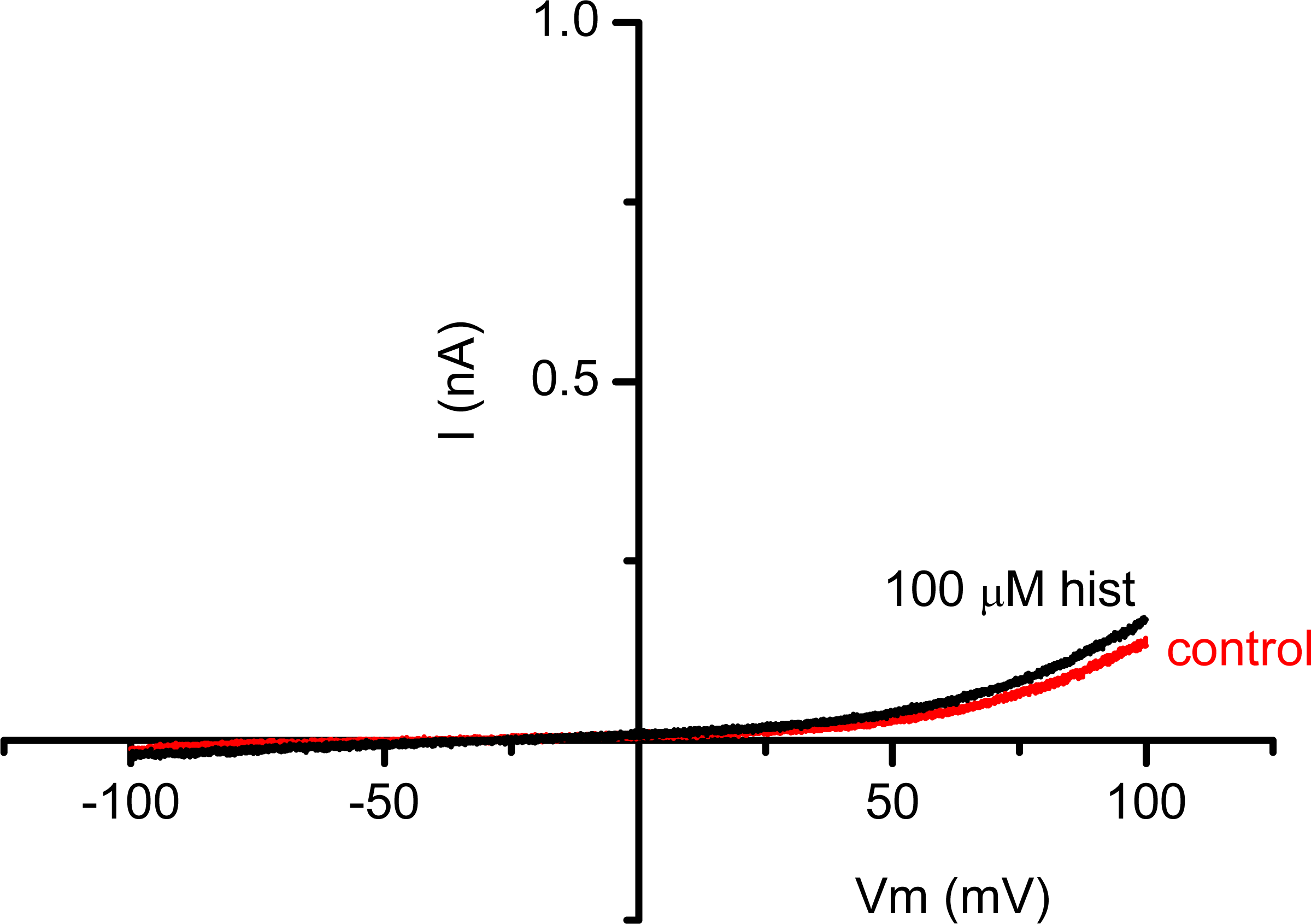

Supplement: Figure S1 — Histamine is unable to evoke whole cell currents in the absence of TRPV1. Current-voltage relationships in HEK293 cells transiently expressing rat H1R (0.2 µg/ml) and GFP (1 µg/ml) proteins before (red trace) and after exposure to histamine (hist) 100 µM (black trace). Data were obtained by voltage ramps from −100 mV to +100 mV with a duration of 1.5 s applied every 5 s. Current was sampled at a frequency of 20 kHz. Series resistance compensation of >50% was used. (TIF) [file pone.0081618.s001.tif]
